# Challenges in the maintenance of an open hospital-based cancer registry system in a low-to-middle-income country (LMIC): 2017–2022 experience

**DOI:** 10.1371/journal.pdig.0000328

**Published:** 2024-01-24

**Authors:** Beatrice Tiangco, Shanaia Esthelle Joy Daguit, Nicole Cathlene Astrologo, Leo Flores, Ric Nonato Parma, Leo Anthony Celi

**Affiliations:** 1 Cancer CARE Registry and Research Philippines Foundation, Inc, Pasig, Philippines; 2 University of the Philippines National Institutes of Health, Manila, Philippines; 3 University of the Philippines Los Baños, Los Baños, Philippines; 4 Laboratory for Computational Physiology, Massachusetts Institute of Technology, Cambridge, Massachusetts, United States of America; 5 Division of Pulmonary, Critical Care and Sleep Medicine, Beth Israel Deaconess Medical Center, Boston, Massachusetts, United States of America; 6 Department of Biostatistics, Harvard T.H. Chan School of Public Health, Boston, Massachusetts, United States of America; Fundación Progreso y Salud: Junta de Andalucia Consejeria de Salud y Familias Fundacion Progreso y Salud, SPAIN

## Abstract

Hospital-based cancer registries (HBCRs) record data on all patients diagnosed and/or treated for cancer at healthcare facilities and evaluate the burden of the disease and the quality of healthcare services at that hospital, helping improve patient care, and providing an assessment of healthcare quality. The CARE PH app was created as a tool to facilitate a system of hospital-based cancer registries in the Philippines, a lower middle-income country. From 2017 to 2022, a total of 60,021 cancer registrants from 44 CARE PH hospitals were entered into the database. Breast cancer was the most common primary site, accounting for 17,660 cases (29.4%). This was followed by colorectal cancer at 11.1%, cervical cancer at 6.2%, head and neck cancer at 5.9%, and prostate and other male genital cancer at 5.1%.Among the 30 data fields collected, 17 exhibited 0–20% missing data, eight displayed 21%-90% missing data, while five depicted 91%-100% missing data. Most of the data fields with missing data are in the treatment and follow-up modules, which are stored in separate forms in a patient’s record. Digital transformation of hospitals from paper-based charts to electronic medical records, and the integration of the HBCR to the EMR and hospital information system, will likely be the best solution for these limitations. It is recommended that the creation and maintenance of HBCRs nationwide must be harmonized, and embedded in all relevant national programs and legislations. The development of an information technology process that is based on a cancer patient’s journey, should be built on an open system embedded in a well designed enterprise architecture, functioning under the guidance of a strong leadership and governance team. All these must be present in order to create and maintain a robust HBCR that is useful for furthering cancer registry and research in the country.

## Author summary

This is a proof-of-concept paper on the challenges in the maintenance of an open disease- specific registry. It tells the story of a hospital-based cancer registry system—its people, process and technology components, the real-life experience of its creation, and the challenges met on the ground, given the required data fields of the registry and the limitations of the current landscape in information technology in a low-to-middle income country (LMIC). The lessons learned are useful as we work towards the global United Nations Sustainable Development Goal of Good Health and Wellbeing by the year 2030, and the local government programs of universal healthcare and cancer control by 2030.

## Introduction

A cancer registry is an information system or database designed for the collection, storage, and management of cancer data from a specified population, which gives its end-users a snapshot of the real-world cancer burden [1]. Although cancer registries were originally used to calculate rates of incidence, and compare risk of various cancers in different populations, developments have helped them evolve to include studies of cancer cause and prevention. Collected registry data now also serve as a primary resource for epidemiological, causality, feasibility and effectivity research, as well as provide critical information needed in planning and evaluating cancer prevention and control interventions [1–3]. There are two major types of cancer registries: population-based registries, and hospital-based registries [1]. Population-based cancer registries (PBCRs) record all cases in a geographically defined population from multiple sources and measure the impact of the disease in specific demographics such as age, gender, etc. PBCRs are designed to determine cancer patterns and trends, guide health policies in surveillance, control and funding, as well as advance clinical, epidemiological, and health services research [3].

On the other hand, hospital-based cancer registries (HBCRs) record data on all patients diagnosed and/or treated for cancer at a particular healthcare facility and evaluate the burden of the disease and the quality of healthcare services at that hospital. The primary focus is on improving patient care at that hospital, as well as on administrative processes, clinical research, and professional education [1,2]. HBCRs are used mainly to provide an assessment of patients’ needs, cancer programs, and health care quality within a health institution [3].

A systematic review of hospital based cancer registries (HBCRs) conducted in 2017 noted that HBCRs are mostly used for management of cancer programs and improving quality of care. Other functions include epidemiological and clinical research, education, policy making, evaluation of implantation of clinical practice guidelines, planning and monitoring of cancer control programs, including prevention, screening, treatment, and palliative care. HBCRs remain an important resource for planning and monitoring of cancer control programs, as they play a role in the improvement of quality of care of cancer patients [4].

Several countries have already established HBCRs, including Japan with 397 HBCRs [5] which provide evidence for clinical measurements and create more accurate health policies for its population. HBCRs have also been established in low- and middle-income countries such as Colombia [6] and Nigeria which has 19 HBCRs [7]. These registries have been useful in improving cancer programs, providing a better understanding of the region’s response capability against cancer, and giving an optimal coverage of cancer data. Such strategies are important in low- to middle-income countries with weak surveillance systems and scarce financial, human, and infrastructural resources for cancer management and control [4–7].

In the Philippines, a lower middle-income country with a population of 109 million, 69.4 million of which are in the 15–64 working age group [8], there is still no centralized population-based cancer registry, but rather separate PBCRs led by local government units in different provinces such as the Department of Health-Rizal Cancer Registry (DOH-RCR), Philippine Cancer Society-Manila Cancer Registry (PCS-MCR), Cebu Cancer Registry (CCR) and Davao Cancer Registry (DCR) [5]. These PBCRs utilize an active method of data collection, to provide information used as the basis for most cancer prevention programs and activities of the Department of Health [9], but these do not often include clinical data, thus limiting the assessment of important variables, such as accuracy of diagnosis, quality of treatment, demand for health services, among others [10].

Although the benefits of implementing an HBCR have been evidenced, it has been observed that its success over time requires interest from the institution, engagement of stakeholders, and financial support. Ethnic variations and environmental influences also make incidence of cancer variable across different populations, hence making it mandatory to have patient databases in every hospital, so that region specific data may be created and policies formulated.

This is a follow-up paper on the Creation and Maintenance of a Hospital-Based Cancer Registry (HBCR) System [11]. The objective of this study is to describe the researchers’ experience in implementing the Cancer CARE Registry and Research Philippines’ (CARE PH) Hospital-based Cancer Registry (HBCR) System in the six years since it started data gathering.

## Methodology

### Implementation and building a database

CARE Philippines (CARE PH) started out as a software tool created in a collaboration between a practicing Medical Oncologist and Epidemiologist, and a Health Information Technology specialist through consultations with different stakeholders such the Department of Health (DOH) Knowledge Management and Information Technology Service (DOH-KMITS), hospital cancer committees, cancer specialists, and cancer researchers, for gathering demographic and clinical information to help the Department of Health determine incidence of disease and prioritize public health activities.

It has since evolved into a loose organization of hospitals (CARE PH Hospitals) and their registry staff that use the CARE PH app for their hospital cancer registry. Each hospital has the CARE PH app embedded into their local area network, kept secure by the hospital IT department or tumor registry office; it has a capacity to share anonymized data with a secure central database. The summary data from all participating hospitals is published in yearly reports presented to the member hospitals, and is also made available in the organization website (https://careph.org).

### Ethics statement and data privacy

The CARE PH HBCR System complies with the Philippine government’s Data Privacy Act of 2012 and was piloted in 2015 at the National Kidney and Transplant Institute (NKTI), a government-owned specialty hospital, and at The Medical City Pasig (TMC), a privately-owned tertiary hospital. CARE PH first submitted its proposal to these hospitals’ ethics regulatory boards which then approved the protocol and informed consent process. Formal written consent was obtained from patients enrolled from the first two hospitals. Subsequent hospital members and their medical directors signed memorandums of agreement with CARE PH where the hospital agreed to use the CARE PH app as their hospital cancer registry and share their anonymized and encrypted cancer registry data with the central CARE PH database which collected only summarized and de-identified registry data.

At the hospital level, all patients need to be informed that the hospital maintains a cancer registry database that contains personal information about those diagnosed with and or treated for cancer within the hospital.

With the passing of Republic Act No. 11215 –the National Integrated Cancer Control Act (NICCA) in 2019 [12], adult and childhood cancers were considered as a notifiable disease in all levels of the healthcare system and required to be reported to the Department of Health. Cancer patients can opt out of the registry by putting in writing their opt out preference, signing and dating such written statements.

### Population and hospital membership

Over the past six years, CARE PH has established Memoranda of Agreement with a total of 44 hospitals with cancer centers, 10 of which are newly onboarded in 2022, as demonstrated in Table 1. Among these, 37 are categorized as tertiary hospitals, five as secondary hospitals, one as a primary care hospital, and one as a standalone cancer center. Geographically, nine of these hospitals are located within the National Capital Region (NCR), while 19 are situated in Luzon (outside NCR), eight in the Visayas, and another eight in Mindanao. Furthermore, the distribution indicates that 15 of these hospitals operate under government management, while 29 are privately owned.

**Table 1:** List of CARE PH hospitals with corresponding level and type of service and bed capacity.

Hospital Name	Level	Type		Bed Capacity
**NCR (n = 9)**				
1. Cardinal Santos Medical Center	Tertiary	Private		245
2. Chinese General Hospital	Tertiary	Private		600
**3. Dr. Jose N. Rodriguez Memorial Hospital and Sanitarium**	**Tertiary**	**Government**		**2,000**
4. East Avenue Medical Center	Tertiary	Government		600
5. Makati Medical Center	Tertiary	Private		600
6. Medical Center Manila	Tertiary	Private		200
7. National Kidney and Transplant Institute	Tertiary	Specialty Government		500
8. Philippine General Hospital	Tertiary	Government		1,500
9. The Medical City Ortigas	Tertiary	Private		600
**LUZON (n = 19)**				
1. Baguio Medical Center	Primary		Government	500
2. Batangas Medical Center	Tertiary		Government	500
3. Bicol Medical Center	Tertiary		Government	500
4. Bicol Regional Training and Teaching Hospital	Tertiary		Government	600
**5. Calamba Medical Center**	**Tertiary**		**Private**	**122**
6. Dagupan Doctors Villaflor Memorial Hospital	Tertiary		Private	125
**7. De La Salle University Medical Center**	**Tertiary**		**Private**	**300**
**8. Divine Grace Medical Center**	**Tertiary**		**Private**	**75**
9. Global Care Cancer Institute	n/a		Standalone	n/a
**10. Mary Mediatrix Medical Center**	**Tertiary**		**Private**	**174**
11. Naga Imaging Center Cooperative Doctors Hospital	Tertiary		Private	99
12. Palawan MMG Cooperative Hospital	Tertiary		Private	80
13. Rizal Medical Center	Tertiary		Government	500
14. Sacred Heart Hospital of Malolos	Secondary		Private	99
15. St. Paul Hospital–Tuguegarao	Tertiary		Private	250
16. The Medical City Clark	Tertiary		Private	100
17. The Medical City Pangasinan	Tertiary		Private	70
18. The Medical City South Luzon	Tertiary		Private	150
**19. Universidad de Sta. Isabel Health Services Department**	**Tertiary**		**Private**	**150**
**VISAYAS (n = 8)**				
**1. AMOSUP-Seamen’s Hospital Iloilo**	**Secondary**		**Private**	**43**
**2. Antique Medical Center**	**Secondary**		**Private**	**152**
3. Iloilo Doctors’ Hospital	Tertiary		Private	300
**4. Metro Iloilo Hospital and Medical Center**	**Secondary**		**Private**	**110**
5. St. Paul’s Hospital of Iloilo	Tertiary		Private	220
6. The Medical City Iloilo	Tertiary		Private	108
**7. Western Visayas Medical Center**	**Tertiary**		**Government**	**400**
**8. Vicente Sotto Medical Center**	**Tertiary**		**Government**	**1,200**
**MINDANAO (n = 8)**				
**1. Ciudad Medical de Zamboanga**	**Tertiary**		**Private**	**160**
**2. Cotabato Regional Medical Center**	**Tertiary**		**Government**	**600**
3. Davao Doctors Hospital	Tertiary		Private	250
4. General Santos Doctors Hospital	Tertiary		Private	202
**5. Metro Davao Medical Research Center**	**Tertiary**		**Private**	**129**
6. Northern Mindanao Medical Center	Tertiary		Government	400
**7. Zamboanga City Medical Center**	**Tertiary**		**Government**	**250**
8. Zamboanga Del Sur Medical Center	Secondary		Government	250
**TOTAL (N = 44)**				

* rows in blue: new in 2022

* rows in bold text: no data shared in 2022

One of the biggest contributing hospital members is the Philippine General Hospital, the National University Hospital, and the national government referral center, which serves more than 600,000 patients from all over the country every year [13]. Ten of the DOH designated cancer care centers (DCCC) are also members of the CARE PH HBCR system including National Kidney and Transplant Institute, East Avenue Medical Center, Bicol Medical Center, Vicente Soto Memorial Medical Center, Western Visayas Medical Center, Batangas Medical Center, Bicol Regional Teaching and Training Hospital, Zamboanga City Medical Center, Northern Mindanao Medical Center and Cotabato Regional and Medical Center [14].

All member hospitals have the following services: pathology, clinical laboratory, diagnostic imaging, hematology, oncology, surgical oncology, chemotherapy, but not all might have radiotherapy. It is notable that only four of the 44 hospitals have a functioning EMR, and most still rely on paper-based charts separate from laboratory information systems (LIS). The CARE PH HBCR System started functioning in July 2017 and its database includes patients with diagnosed cancer from January 13, 2017.

### Case definition

All patients of any age and sex, seen in any hospital part of the CARE PH System or in hospital-affiliated oncology clinics, who have a clear basis for the diagnosis of cancer are included in the registry. Cancer patients undergoing any treatment modality, including palliative care, for the said disease are also included. The main basis for diagnosis is the pathology report of the diagnostic biopsy. In cancers like germ cell tumors where clinical diagnosis using biomarkers, or hepatocellular cancer where imaging of the liver of patients with risk factors like hepatitis B or C, surgical pathology or cytology reports are not needed for diagnosis of cancer and the patient is still entered into the cancer registry.

### Staffing and training

Comprising a Tumor Registry Office in each CARE PH hospital is at least one Doctor Champion, and at least one Tumor Registrar, who are full-time employees of the hospital voluntarily contributing to the local cancer database. The Doctor Champion is an oncologist or general physician who consults regarding the important dates in the patient’s cancer journey and helps the tumor registrar in deciding when the patient was diagnosed, relapsed, progressed, etc., as well as how to categorize the patient’s malignancy. The Tumor Registrar, any hospital staff with a background in healthcare chosen by the Doctor Champion, is trained in Good Clinical Practice and data entry into the cancer registry app and also makes rounds to the different catchment areas within the hospital to collect information and regularly shares the hospital cancer registry data with the central database. Newly onboarded hospitals in the CARE PH HBCR System undergo a one-day training conducted by CARE PH Registry staff, introducing the application to both the Doctor Champion and Tumor Registrar, and are given a training manual which includes step-by-step instructions for encoding and data collection.

### Data sources

The cancer patient journey typically begins with the diagnosis of cancer based on a biopsy report generated in a hospital’s Pathology department. It is worth noting that in the Philippines, patients often receive care in more than one hospital during their cancer journey. Once a cancer diagnosis is confirmed, comprehensive staging procedures, such as radiological imaging and serum biomarker assessments, are conducted to evaluate the extent of the disease and predict its progression. Following this, the diagnosis is typically communicated by an attending oncologist who discusses treatment options, which are made after reaching consensus in a collaborative consultation involving a multidisciplinary healthcare team or hospital tumor board. Evidence-based surgical, radiation, or pharmaceutical interventions are then initiated and continued in a strategic manner with cure, extension of life, or quality of life as outcomes of interest.

Given this overview, catchment areas were identified within each member hospital, including: (1) Records Section, (2) the Pathology Department, (3) Specialty Out-Patient Clinics for cancers such as Germ-Cell Tumors, Liver Cancers, and Brain Cancers which do not need tissue biopsies for clinicians to diagnose and treat as cancer, and (4) Radiation and Chemotherapy Clinics for patients diagnosed in a different hospital, but sought treatment at a CARE PH hospital.

### Data collection

The hospital tumor registrar goes to all the catchment areas, gathers the patients’ identification numbers, and manually enters information into the hospital cancer registry on a regular basis. The data fields are distributed into the following modules: Hospital Information, Patient Data, Cancer Data, Cancer Treatment Intervention, and Patient Current Status Data. The data fields collect both standardized data through pre-selected drop-down options (i.e. primary site, diagnostic coding, staging, status) and free text data, where appropriate (i.e. CARE PH registry number, Patient Identification Number (PIN), imaging or pathology reports). Cancer diagnostic coding is done according to the International Classification of Diseases for Oncology, third edition (ICD-O-3) [15], and the staging is done using the American Joint Committee on Cancer, eighth edition (AJCC 8th) [16].

Only the tumor registrar and select hospital staff who have been properly trained on data entry, handling and security are given usernames and passwords to access the database.

**Fig 1:**
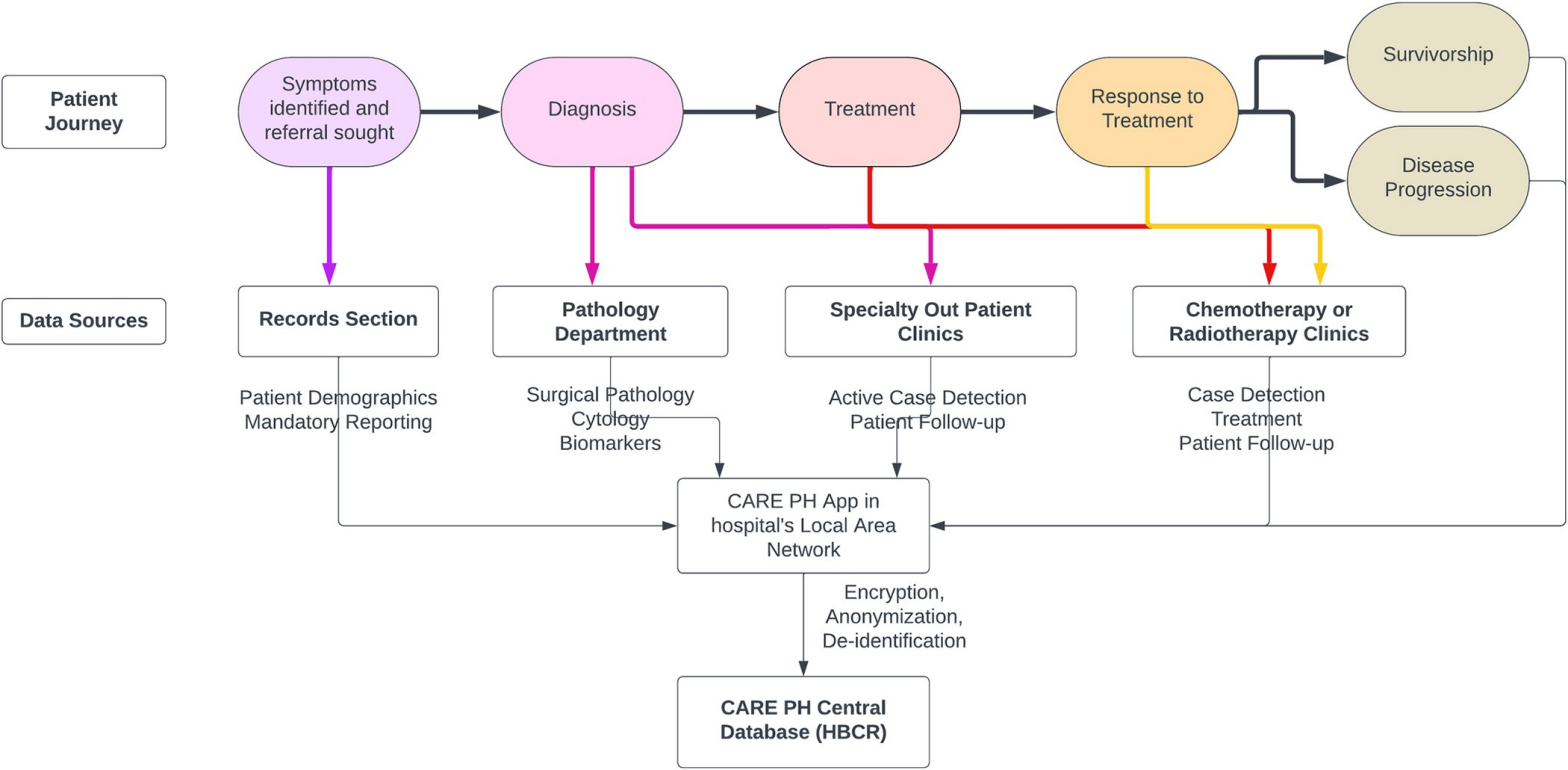
CARE PH Hospital Cancer Registry Catchment areas and data flow.

Fig 1 illustrates the flow of data, mapping the cancer patient’s journey to the entry points of data gathering from the catchment areas to the CARE PH web application, then on to the central CARE PH database. Once member hospitals have completed data collection from their respective sites, data is uploaded to CARE PH central servers on a weekly or monthly basis. The data entered and stored in the local area network includes patient identifiers, but the data shared with the central CARE PH database is encrypted, anonymized and de-identified, where patients are referred to only with a CARE PH registry ID bearing patient initials and birthdate. Registrants in the central database with the same initials, birthdate and primary site but entered into the database by different CARE PH hospitals are red-flagged by the central CARE PH app. These two entries are assumed to be the same patient who transferred from one CARE PH hospital to another, and the number of such entries are presented in the annual report.

After enrollment to the registry, the patient status is checked by the doctor champion and tumor registrar every 6 months or less, if there is any noted status change such as remission, recurrence, relapse or death. Data regarding follow-up status is recorded in the follow-up status and clinical outcome module.

### Quality control and maintenance

There is no standard method for quality control of information for the HBCR. Practices per hospital may vary, with the doctor champion reviewing the information recorded, verifying consistency of data and checking for possible errors in encoding and tumor identification and classification. At the central database level, the CARE PH team works on performing checks to ensure collection of authentic and valid data, as well as checking for possible duplicates.

Regular backups of data are done to ensure no data loss. Backed up data is downloaded and stored in a separate local server and storage device.

### Yearly review and reporting

The yearly report includes a consolidated cancer census that looks at the total number of new registrants, top primary sites for the entire HBCR system and a breakdown per hospital. Age distributions per primary site and top 10 primary sites per age group are also analyzed.

### Analysis

The study primarily employed a descriptive statistical analysis approach, utilizing a dataset spanning six years from 2017 to 2022. An interactive graphing library called Plotly for Python was employed to create graphical representations of various analyses. To facilitate coding and script development, Visual Studio Code, run using Anaconda Navigator, was chosen, ensuring an efficient workflow throughout the process.

To ensure data integrity, an assessment of data fields with missing values was performed. This process contributed to refining the dataset by systematically removing fields exhibiting more than 90% missing values, ensuring that only informative variables were retained for subsequent analysis. Possible replications, for patients who were entered multiple times by different CARE PH hospitals, were removed by matching the automatically assigned registry number containing patient initials and date of birth, with cancer primary site. Following this preprocessing, an overview of cancer registrants in the database was presented, including their distribution across various hospitals and the frequency distributions of primary cancer sites recorded in the CARE PH census. It also explored the ten most common primary cancer types across ten CARE PH-HBCR sites to understand prevailing incidence trends. Additionally, the study examined the age distribution of patients with these top ten primary cancer sites based on data from the CARE PH registry. To offer a broader context, it investigated the regional distribution of cancer registrants, aiming to uncover potential geographic disparities. Furthermore, the analysis included a review of the frequency distribution of staging information across all primary cancer sites to assess the extent of cancer progression at the time of diagnosis.

## Results

The data that support the findings of this study are openly available in figshare at http://doi.org/10.6084/m9.figshare.23665113.

### Data fields in the CARE PH application

The CARE PH application comprises five sections, i.e., Hospital Information, Patient Data, Cancer Data, Cancer Treatment Intervention, and Patient Current Status Data, where each data field is identified by a unique code as detailed in Table 2. Among the 30 data fields, 17 exhibit 0–20% missing data (highlighted in green), eight displayed 21%-90% missing data (highlighted in yellow), while five depicted 91%-100% missing data (highlighted in red).

**Table 2:** Breakdown of null values in the data fields of CARE PH registry.

Entry	Null Count	Total Count	Null Percentage
**HOSPITAL INFORMATION**			
**Date Created**	2	60021	0.0033%
**Institution ID (Internal ID assigned to the Institution or Hospital)**	75	60021	0.12%
**Institution Name (Name of the Institution or Hospital)**	75	60021	0.12%
**Copy of Institution ID**	0	60021	0
**PATIENT DATA**			
**ID (Record ID in the database table)**	0	60021	0
**Hospital Patient ID**	0	60021	0
**Registration ID (Assigned to the Patient in the Institution or Hospital)**	0	60021	0
**Birthdate**	9861	60021	16.43%
**Sex**	80	60021	0.13%
**City**	34149	60021	56.90%
**Provincial Code (Assigned to the province of the patient)**	1230	60021	2.05%
**Patient Date Created (Date when the patient data was first encoded)**	0	60021	0
**CANCER DATA**			
**Primary Site**	0	60021	0
**Primary Site ICD 10 ID (ID assigned to a Primary Site)**	0	60021	0
**ICD 10 ID (ID assigned to a ICD10)**	1608	60021	2.68%
**Health Facility Entry Date (Date when the Patient was first admitted in the Institution or Hospital)**	0	60021	0
**Incidence Date**	13823	60021	23.03%
**Pathology Report No**	18389	60021	30.64%
**Stage**	44717	60021	74.50%
**Patient Primary Site Date Created (Date when the Primary Site data was first encoded)**	0	60021	0
**CANCER TREATMENT INTERVENTION**			
**Cancer Surgery Date**	60021	60021	100%
**Radiation Treatment Start Date**	60021	60021	100%
**Systemic Treatment Start Date**	60021	60021	100%
**Palliative Treatment Start Date**	60021	60021	100%
**PATIENT CURRENT STATUS DATA**			
**Patient Primary Site Date Updated (Date when the Primary Site data was last updated)**	0	60021	0.0
**Primary Site Change in Status Stage**	55711	60021	92.82%
**Patient Primary Site Status Date Created (Date when the status of the primary site was first encoded)**	48726	60021	81.18%
**Patient Primary Site Status Date Updated (Date when the status of the primary site was last updated)**	48727	60021	81.18%
**Patient Primary Site Status Date (Date of the Primary Site Change in Status)**	48737	60021	81.20%
**Patient Primary Site Status Change (Primary Site Change in Status (incidence, no change, remission, stable disease, etc.))**	49034	60021	81.69%

* rows in green: 0%-20% missing data

* rows in yellow: 21%-90% missing data

* rows in red: 91%-100% missing data

Data fields showing missing data within the 21–90% range were observed in the Patient Data section ("City"), the Cancer Data section ("Incidence Date," "Pathology Report Number," and "Stage"), and the entirety of the Patient Current Status section. The data field “City” holds no relevance for data analysis as data is analyzed by region and not city of residence; consequently, this particular field will be removed from the application in future updates. Within the data fields highlighted in yellow, namely “Incidence Date”, “Pathology Report Number” and “Stage”, the presence of missing data can be attributed to those patients who have undergone diagnostic biopsies and staging procedures done in healthcare facilities outside the CARE PH hospital from where they are receiving cancer treatment. While these external pathology reports, staging laboratory, and radiologic images may be in their oncologists’ clinic charts, these remain inaccessible to the hospital tumor registrar.

Meanwhile, data fields exhibiting over 90% missing data were found exclusively within the Cancer Treatment Intervention section and in the “Primary Site Change in Status Stage” data field of the Patient Current Status section. This missing data, evident in the red-highlighted rows of the Cancer Treatment Intervention section, underscores the inherent challenge of accessing the clinical notes maintained by the treating physicians.

### Hospital membership

Out of the 44 hospital institutions that signed a Memorandum of Agreement with CARE PH, 27 CARE PH-affiliated hospitals actively contributed their data to the central CARE PH database, while 17 CARE PH hospitals did not participate in data sharing. Their lack of participation can be attributed to several reasons, including one of the following: (1) the inclusion of new members who were not yet prepared to share their data [17], (2) temporary halts in data collection by some hospitals due to force majeure or pandemic-related challenges, resulting to difficulties in resuming [17], and (3) a subset of hospitals opting to input their data into the non-communicable diseases registry of the Department of Health (DOH) instead of the CARE PH database.

In the period spanning from 2017 to 2022, a total of 60,021 cancer registrants were entered into the CARE PH central database, with the Philippine General Hospital having the highest number of registrants, and National Kidney & Transplant Institute having the second highest number of cancer registrants across all partner hospitals, as seen in Table 3. NCR harbors a higher proportion of partner hospitals compared to other regions, potentially suggesting a possibility of expanding hospital partnerships beyond the current hospital institutions.

**Table 3:** CARE PH hospitals with the number of registrants in database.

Region	Institution (year joined)	Number of Registrants
NCR	1. Philippine General Hospital	12,040
	2. National Kidney & Transplant Institute	11,244
	3. The Medical City Ortigas	9,845
	4. Makati Medical Center	3,940
	5. Medical Center Manila	2,862
	6. Chinese General Hospital	2,762
	7. East Avenue Medical Center	2,290
Region I	1. Dagupan Doctors Villaflor Memorial Hospital	5,646
	2. The Medical City Pangasinan	69
CAR	1. Baguio Medical Center	131
Region II	1. St. Paul Hospital of Tuguegarao	133
Region III	1. The Medical City Clark	237
	2. Bulacan Sacred Heart	179
Region IV-A	1. Batangas Medical Center	1,412
	2. Rizal Medical Center	935
	3. Global Cancer Care Institute	14
Region IV-B	1. Palawan MMG-PPC	3
Region V	1. Bicol Medical Center	2,474
Region VI	1. The Medical City Iloilo	283
	2. Iloilo Doctors Hospital	239
Region IX	1. Zamboanga Del Sur Medical Center	13
	2. Zamboanga City Medical Center	5
Region X	1. Northern Mindanao Medical Center	1,161
Region XI	1. Davao Doctors Hospital	736
	2. Metro Davao Medical and Research Center	57
Region XII	1. Cotabato Regional & Medical Center	1,207
	2. General Santos Doctors Hospital	29
Missing Values		75
TOTAL		60,021

### Cancers diagnosed and/or treated in hospitals

**Fig 2:**
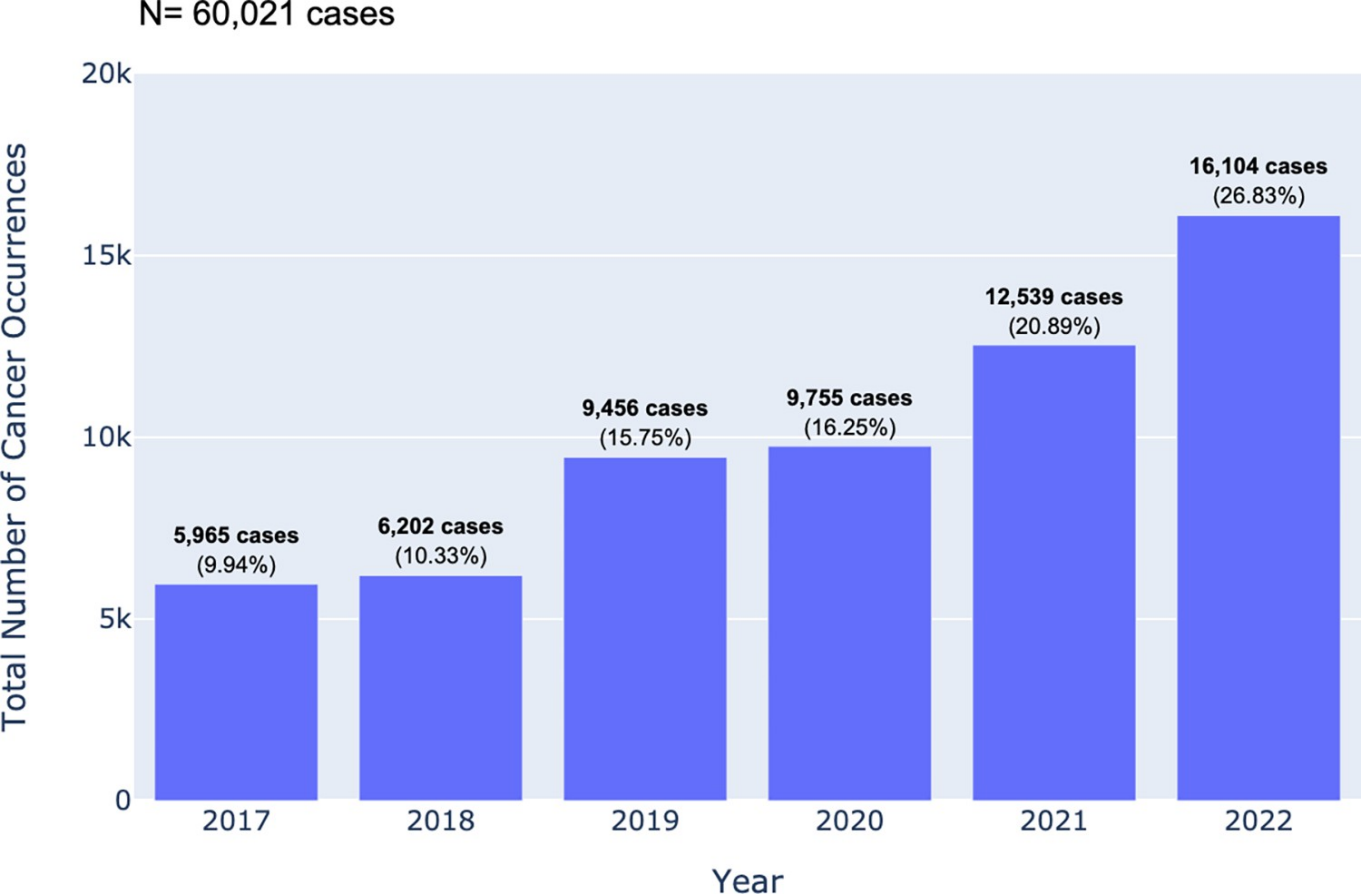
Frequency of primary cancer sites in CARE PH cancer census from 2017 to 2022.

**Fig 3:**
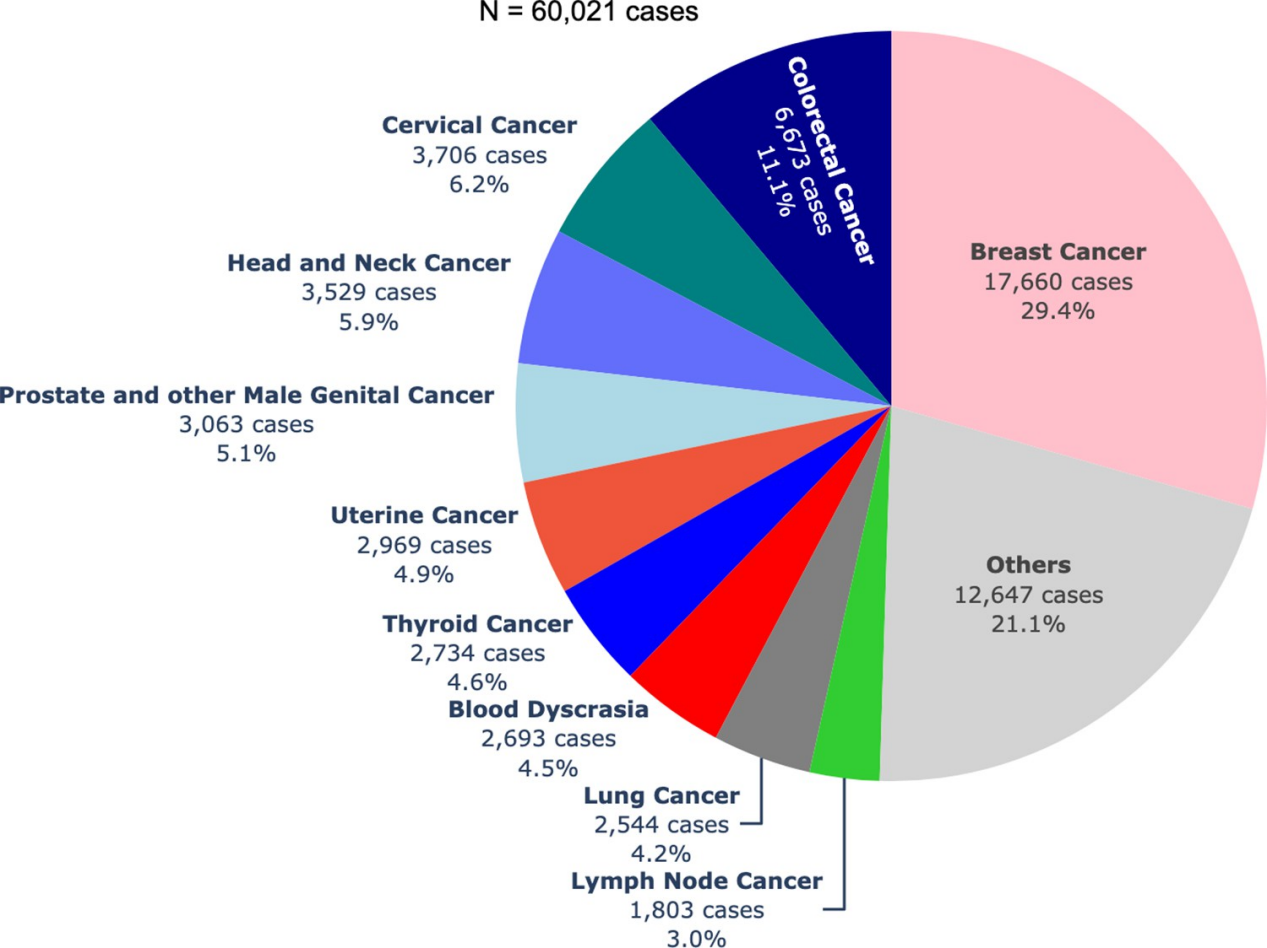
Frequency of top ten primary cancer sites in CARE PH Cancer Census from 2017 to 2022.

**Fig 4:**
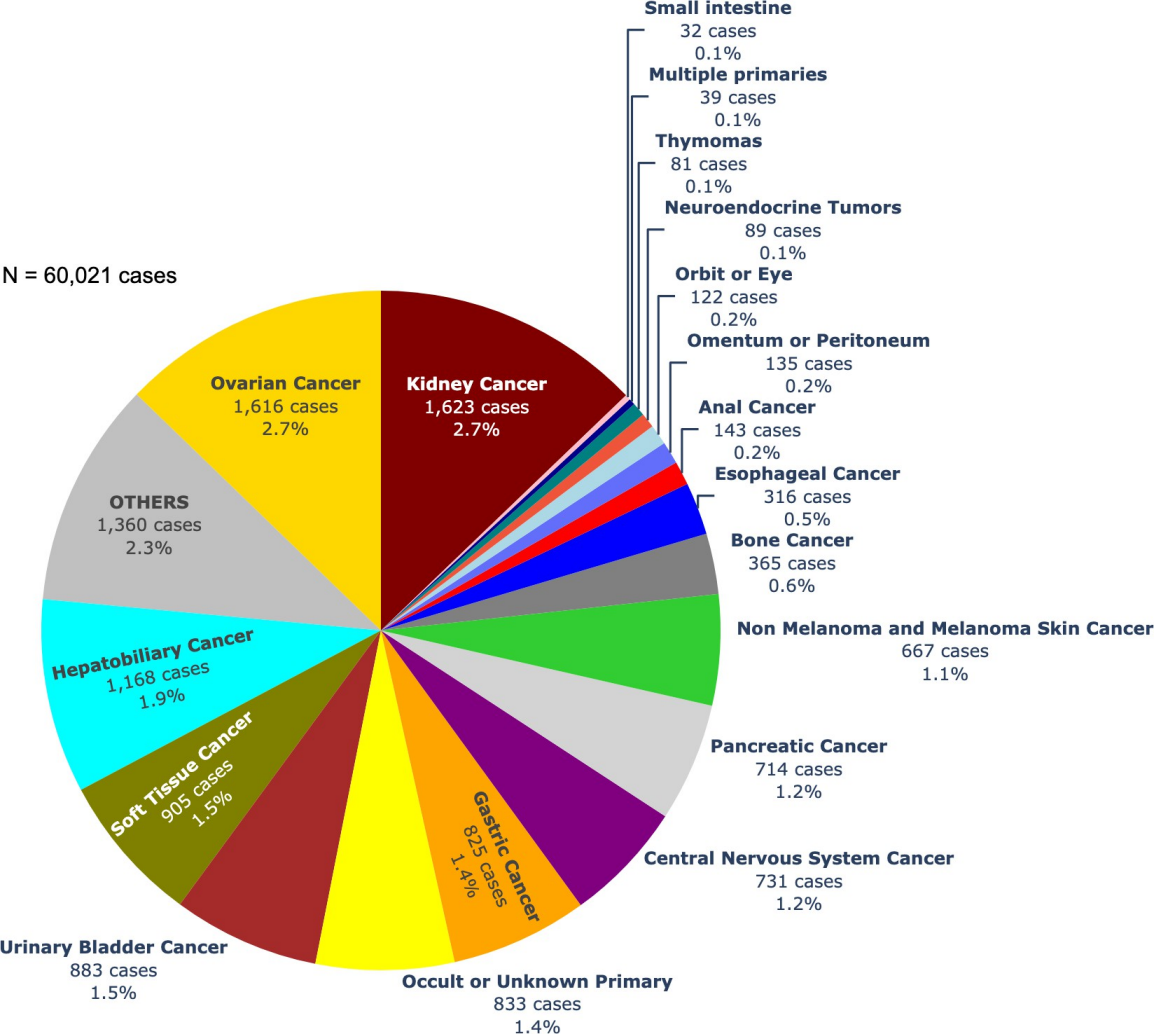
Frequency of 11th-28th ranking primary cancer sites in CARE PH Cancer Census from 2017 to 2022.

The number of diagnosed cancer cases over the years encompasses a range of 28 possible primary sites with corresponding ICD-10 categories in the CARE PH app. The progressive increase in the number of newly diagnosed cancer cases as depicted in Fig 2, can be related to the increase in the number of hospitals transmitting data. Among the 60,021 cancer patients with recorded primary sites in the central CARE PH database from 2017–2022, breast cancer was the most common primary site, accounting for 17,660 cases, or 29.4% of all cancer registrants (Fig 3). This was followed by colorectal cancer at 11.1%, cervical cancer at 6.2%, head and neck cancer at 5.9%, prostate and other male genital cancer at 5.1%, uterine cancer at 4.9%, thyroid cancer at 4.6%, blood dyscrasia at 4.5%, lung cancer at 4.2%, and lymph node cancer at 3.0%. There were 12,647 patients with cancers from less common primary sites. Fig 4 shows the breakdown of these other cancers.

On another note, Table 4 demonstrates the frequency distribution for the most prevalent primary cancer cases diagnosed at the top 10 CARE PH-HBCR sites, offering insights into regional disparities in cancer diagnosis and healthcare accessibility. A significant concentration of cancer diagnoses occurred within NCR, home to some of the Philippines’ leading healthcare institutions. The roles of these prominent hospitals are pivotal in cancer diagnosis, reflecting both the high population density in the NCR and the advanced medical infrastructure these institutions offer. Beyond NCR, several hospitals outside this region also emerged as frequent sites for cancer diagnosis. Notably, Dagupan Doctors Villaflor Memorial Hospital, Bicol Medical Center, and Batangas Medical Center have made their mark as key players in addressing the cancer burden in their respective areas. This highlights the crucial role of regional healthcare centers in providing essential cancer diagnosis and treatment services to communities beyond the NCR.

**Table 4:** Frequency distribution of top ten primary cancers at ten CARE PH-HBCR sites.

	*Breast Cancer*	*Colorectal Cancer*	*Cervical Cancer*	*Head and Neck Cancer*	*Prostate and other Male Genital Cancer*	*Uterine Cancer*	*Thyroid Cancer*	*Blood Dyscrasia*	*Lung Cancer*	*Lymph Node Cancer*	*Other Primary Sites*	*TOTAL*
*Philippine General Hospital*	2359	1316	970	710	326	1008	733	517	386	382	3333	12040
*National Kidney & Transplant Institute*	2382	1019	283	284	1246	172	112	1566	384	534	3262	11244
*The Medical City Ortigas*	3205	989	730	568	520	598	668	267	540	178	1582	9845
*Dagupan Doctors Villaflor Memorial Hospital*	1908	604	622	634	116	254	278	24	332	104	770	5646
*Makati Medical Center*	1399	373	147	164	173	205	250	105	287	131	706	3940
*Medical Center Manila*	1052	366	139	193	90	123	207	27	153	51	461	2862
*Chinese General Hospital*	765	413	128	128	282	171	220	3	66	18	568	2762
*Bicol Medical Center*	763	254	333	211	81	179	39	62	77	47	428	2474
*East Avenue Medical Center*	844	436	57	297	55	45	55	3	42	79	377	2290
*Batangas Medical Center*	556	223	120	74	18	74	25	38	35	30	219	1412
*Other hospital institutions*	2387	678	177	266	156	139	146	80	241	249	912	5431
*Null Count*	40	2	0	0	0	1	1	1	1	0	29	75
**TOTAL**	17660	6673	3706	3529	3063	2969	2734	2693	2544	1803	12647	60021

Table 5 presents a breakdown of the age distribution of the top ten primary cancer sites, where the ages were retrieved by referencing the date of initial diagnosis of the primary sites. The cells highlighted in yellow indicate the age range with the highest number of registrants. Breast cancer, head and neck cancer, uterine cancer, and thyroid cancer were the most frequently diagnosed types of cancer among individuals aged 50–59 years, while colorectal cancer, prostate and other male genital cancer, blood dyscrasia, lung cancer, and lymph node cancer were more commonly diagnosed in individuals aged 60–69 years. Cervical cancer was most frequently diagnosed in individuals aged 40–49 years. These insights denote the significance of age-specific cancer screening to address the varying prevalence of cancer types across different age groups, ultimately contributing to improved healthcare outcomes and early interventions.

**Table 5:** Age distribution of top 10 primary cancer sites from CARE PH registry.

PRIMARY CANCER SITE	AGE DISTRIBUTION								Missing values	TOTAL
	0–19	20–29	30–39	40–49	50–59	60–69	70–79	Over 80		
**Breast Cancer**	102	171	1376	3735	4397	3166	1209	287	3217	17660
**Colorectal Cancer**	54	98	329	695	1445	1902	925	235	990	6673
**Cervical Cancer**	29	96	567	868	768	480	143	25	730	3706
**Head and Neck Cancer**	78	150	302	561	751	723	309	87	568	3529
**Prostate and other Male Genital Cancer**	16	76	38	68	297	993	833	222	520	3063
**Uterine Cancer**	10	40	228	441	792	616	221	23	598	2969
**Thyroid Cancer**	50	230	346	374	489	404	135	38	668	2734
**Blood Dyscrasia**	357	274	249	314	405	497	264	66	267	2693
**Lung Cancer**	18	24	60	192	394	727	466	122	541	2544
**Lymph Node Cancer**	129	310	243	174	241	324	160	43	179	1803
**Others**	496	541	894	1629	2683	2892	1532	397	1583	12647
**TOTAL**	1339	2010	4632	9051	12662	12724	6197	1545	9861	60021

Legend

Yellow highlighted cells indicate the age range with the highest number of registrants

Out of the total population, 1339 (2.2%) belong to the pediatric population (aged 0–19). The most frequently diagnosed cancers under this group are blood dyscrasias, with 357 cases or 26.7% of the population, followed by lymph node cancer at 9.6%, breast cancer at 7.6%, head and neck cancer at 5.8%, and colorectal cancer at 4.0%. Four hundred ninety-six fall under the “Others” primary site, which is a basket category of primary sites which cannot be classified under the other categories, including carcinoma-in-situ, melanoma-in-situ, and neoplasms of uncertain and unspecified behavior.

**Fig 5:**
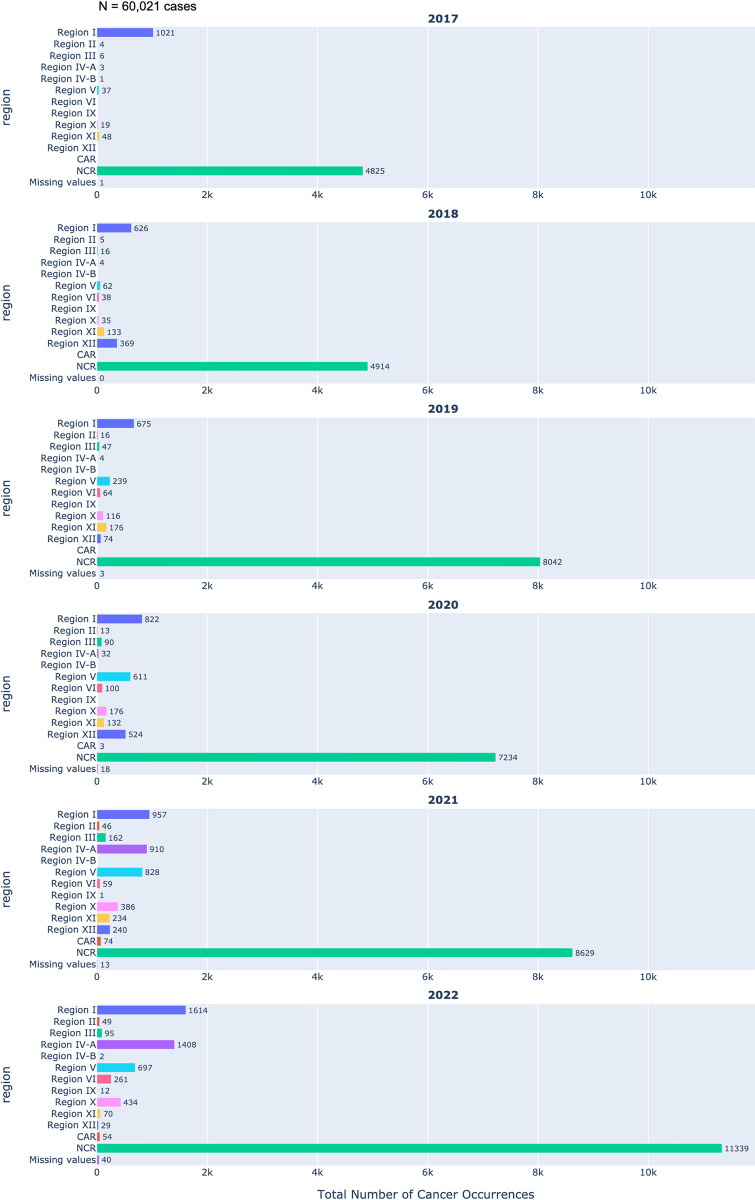
Regional distribution of cancer registrants 2017–2022.

In 2017, only three hospitals were actively sharing their data for the CARE PH hospital cancer registry. Specifically, two of these hospitals were located in the National Capital Region (NKTI and The Medical City Ortigas), while the third was situated in Region I (Dagupan Doctors Villaflor Memorial Hospital). Over the subsequent years, there has been a steady increase in the number of participating hospitals, reflected in the progressive increase of yearly registrants, with representation expanding to encompass various regions. Remarkably, the National Capital Region consistently exhibited the highest annual contribution of cancer patient data (as illustrated in Fig 5). As of the year 2022, hospitals from Regions I, II, III, IV-A, IV-B, V, VI, IX, X, XI, XII, Cordillera Administrative Region (CAR), and NCR actively contribute to the CARE PH hospital cancer registry. In contrast, Regions VII (Central Visayas), VIII (Eastern Visayas), and Autonomous Region of Muslim Mindanao (ARMM) have yet to establish their presence within the CARE PH HBCR system. This could be because Region VII, Cebu maintains its own population-based cancer registry, directly transmitting cancer data to the Department of Health. Moreover, Region VIII experienced significant challenges following the impact of typhoon Yolanda in November 2013, leading to the departure of specialist oncologists who have not returned to the region. Additionally, ARMM, situated in the southern part of the archipelago, faces irregular and challenging internet connectivity, thereby contributing to the missing data.

The increasing participation of hospitals in the CARE PH hospital cancer registry, from three in 2017 to multiple regions by 2022, underscores a growing commitment to comprehensive cancer data collection and highlights the need for further expansion to ensure a more holistic understanding of cancer prevalence nationwide.

### Baseline cancer staging

**Fig 6:**
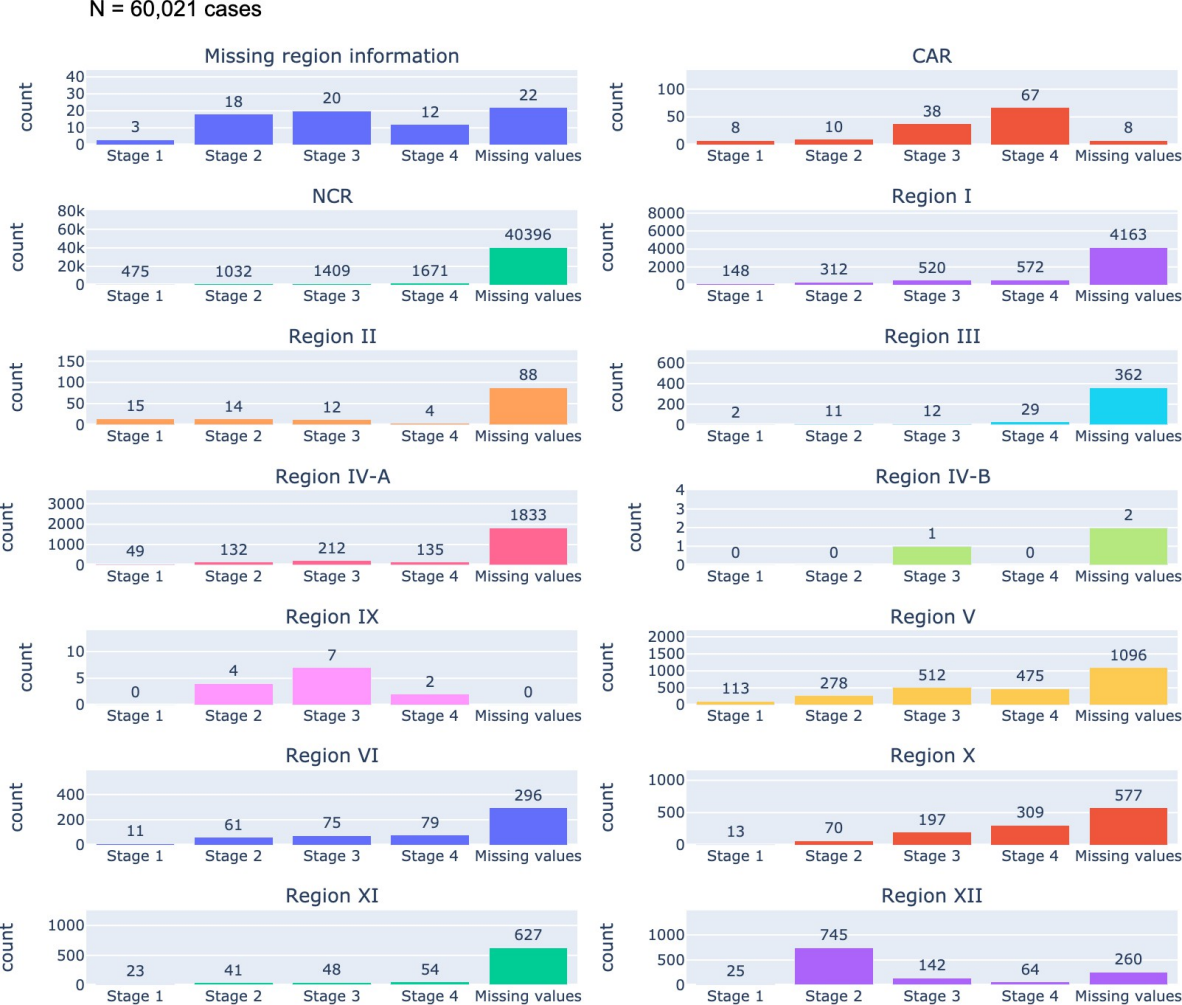
Frequency distribution of staging information from all primary cancer sites in CARE PH Cancer Census from 2017 to 2022.

Fig 6 showed that 49,730 or 83% of all 60,021 patients in the database had missing data in the “stage of baseline cancer” data field. Cancer stage is considered essential in the diagnosis of any cancer as it provides information regarding tumor burden and is a prognostic indicator for the disease. The cancer staging system that is most commonly used globally is the TNM Classification system where T refers to tumor size, N refers to regional lymph node involvement and M refers to presence or absence of metastases. In Fig 6, the missing values bar encompasses primary cancer sites including blood cancers, germ cell tumors, and brain tumors, which are not amenable to TNM classification, and it also accounts for entries where staging information remains unknown to the data encoder.

**Fig 7:**
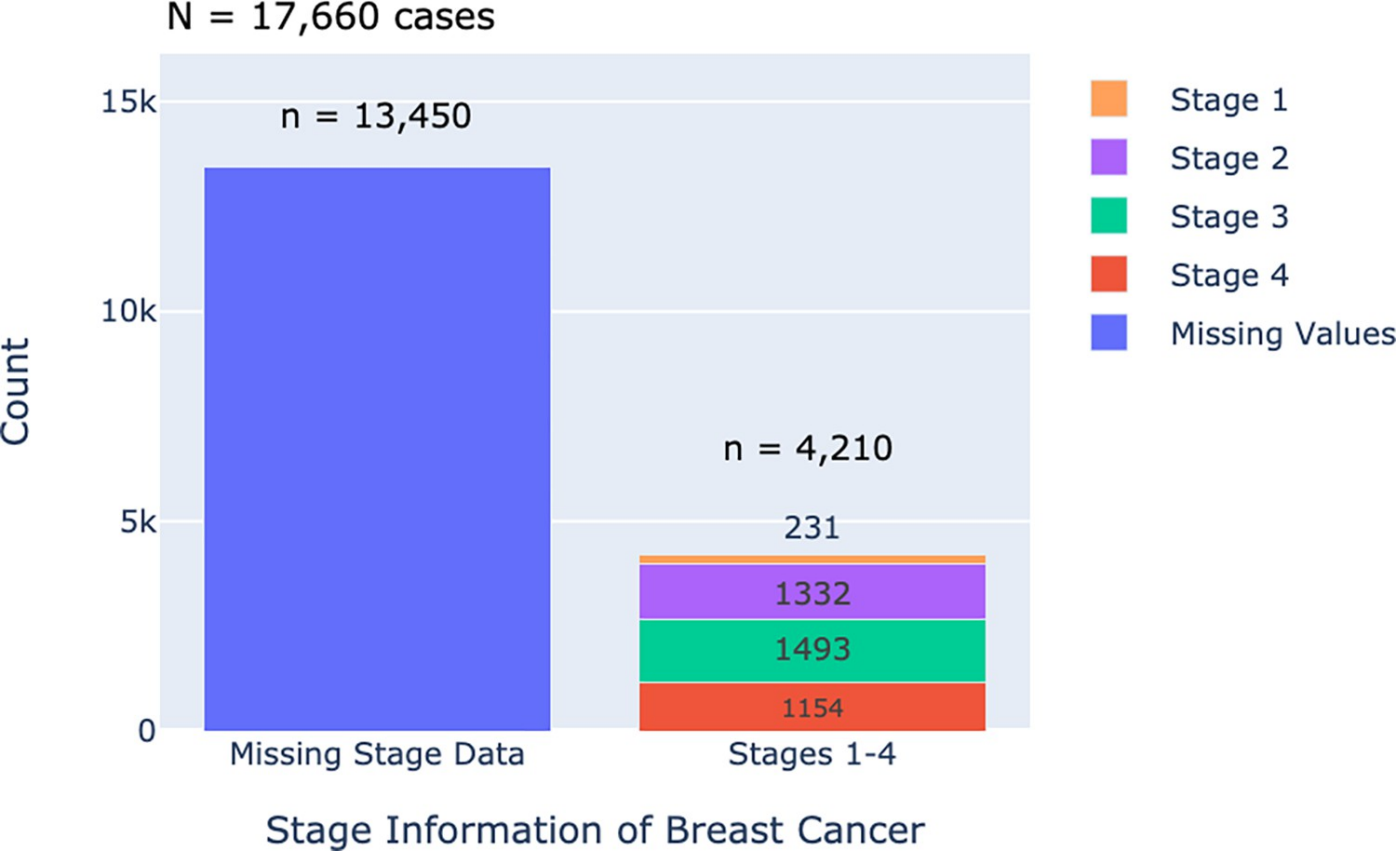
Stage information of breast cancer.

Distribution of cancer staging varies across regions, yet uniformly, all regions exhibited a higher prevalence of cancers in stage 3 or 4 in comparison to stages 1 and 2 upon initial entry into the hospital cancer registry. A noticeable scarcity of stage 1 diagnoses is seen, a phenomenon that ideally should be more pronounced with the effective implementation of cancer screening programs. A similar trend is seen when examining the most prevalent cancer type within the 2017 to 2022 CARE PH census. As depicted in Fig 7, breast cancer in the Philippines is most often diagnosed in Stage 3, followed by Stage 2, Stage 4, then stage 1.

## Discussion

This paper presents an exploratory data analysis conducted on a cohort of 60,021 cancer patients who have been enrolled in the central database of the CARE PH-HBCR from 2017 to 2022. CARE PH-HBCR constitutes a collaborative network encompassing 44 hospitals within the Philippines, comprising 15 publicly funded institutions and 29 privately operated hospitals. Out of the 44 hospital institutions, 27 hospitals actively and regularly contributed de-identified patient data at regular intervals, typically on a monthly to quarterly basis, through the utilization of the sole web-based HCBR system currently available in the country.

### Cancer incidence

In light of the Global Cancer Observatory’s 2020 population-based cancer registry data, the estimated annual incidence of cancer in the Philippines stands at 153,751 cases [18]. In contrast, the CARE PH HBCR network recorded 16,708 diagnosed and/or treated cancer patients in 2022. While this figure is not fully representative of cancer incidence or newly diagnosed cases, as per the PBCR definition, it does represent a little over 10% of the annual estimated incidence of newly diagnosed cancer cases.

When examining the epidemiology of cancer in the country, the regional data presented in the database needs to be interpreted with caution. Not all regions are adequately represented, and the regions that are included have varying starting dates of data collection. For instance, analysis of the data presented in Table 3 highlights that NCR harbors the highest proportion of partner hospitals when compared against other regions. This disparity in hospital distribution potentially results in a situation where the reported cancer cases may not sufficiently reflect the entirety of the country’s various regions. Furthermore, as depicted in Fig 2, some have been collecting data since as early as 2017, while others may have only started entering data in the latter half of 2022. As a result, the overall analysis may be constrained in terms of its completeness and accuracy.

While there are caveats in observing the regional data, the general trend of cancer cases from 2017 to 2022 reveals a consistent increase in the number of member hospitals and consequently, in the number of patients with cancer registered. However, an observable plateau in these figures became evident in 2020, coinciding with the onset of the COVID-19 pandemic. As seen in Fig 2, the pandemic-induced disruption had a notable impact on the diagnosis and treatment of cancer in the Philippines, resulting in a significant deficit in expected numbers of people with cancer to undergo diagnostic and therapeutic modalities in hospitals. In 2021, a resurgence in the number of hospitals occurred, and the count of cancer patients accessing these healthcare facilities eventually caught up with the preceding upward slope of trajectory. It is worth postulating that there were relatively more Stage 2 and 4 patients diagnosed and/or treated in CARE PH hospitals in 2021, relative to that number in 2019.

A study conducted in the United Kingdom revealed significant increases in avoidable cancer-related deaths in England, attributable to diagnostic delays resulting from the impact of the COVID-19 pandemic [19]. Following the implementation of a national lockdown in 2020 in response to the pandemic, several measures were enacted, including the suspension of cancer screening, deferral of routine diagnostic procedures, and prioritization of urgent symptomatic cases, similar to the occurrences in Spain [20], Canada [21], Brazil [22], the United States [23], and the Netherlands [24]. These findings collectively suggest that the observed trends in 2020, aligning with the COVID-19 pandemic, could be linked to the disruptions caused by the pandemic across healthcare systems globally.

Regardless, the rising number of cancer registrants in the past six years reflects an increasing trend of partner hospital institutions sharing their data in the CARE PH central database. Such a development indicates the registry’s growing comprehensiveness and accuracy, providing a foundation for a more detailed understanding of cancer burden in the Philippines–a necessary step in developing effective cancer control strategies and programs, for better health outcomes.

### Baseline cancer staging

In 2020, a significant shift occurred as breast cancer, by a narrow margin, surpassed lung cancer in terms of incidence, according to data from the Global Cancer Observatory’s population-based cancer registry [11]. In the Philippines, specifically within the population-based cancer registries of Rizal Province, Metro Manila, and Cebu, breast cancer has always far outnumbered the incidence of lung cancer in incidence as depicted in Fig 3. This phenomenon is not unique to the Philippines; across much of Asia, breast cancer has held its position as the most frequently diagnosed cancer for many years [25–27].

Focusing on breast cancer staging data, we gain deeper insights into this prevailing health concern. As depicted in Fig 7, we find that 13,450 out of 17,660 patients, representing 76% of cases, lack essential staging data. From the 24% of patients with staging information, it can be observed that in the Philippines, breast cancer diagnoses are predominantly made at advanced stages, with Stage 3 cases constituting the highest proportion at 35.5%, followed closely by Stage 2 at 37%, and Stage 4 at 27.5%. In contrast, Stage 1 breast cancer is notably less common, indicating a pressing need for increased efforts in early detection and awareness campaigns to identify cases at earlier, more treatable stages.

In demonstrating the significance of executing screening programs to reduce incidence of advanced-stage breast cancers, in countries like Lebanon, the implementation of screening programs has yielded notable results, with approximately 31% of breast cancer patients being diagnosed at stage 1, 47% at stage 2, 14% at stage 3, and 8% with distant (metastatic) stage 4 disease [28]. In the Münster district of northwestern Germany, carrying out an organized mammography screening program resulted in a significant decrease in the incidence rates of advanced breast cancer, while simultaneously leading to an increase in early-stage breast cancer incidence rates [29]. A study utilizing data from the the United States Surveillance, Epidemiology, and End Results Program reported that the incidence of advanced breast cancer would have been 29% higher in the absence of mammography screening [30]. The aforementioned studies serve as valuable baselines for assessing the effectiveness of breast cancer screening initiatives, with the goal of being able to diagnose more non-palpable breast masses in the earlier stages of cancer. Comparative data like these, which show the baseline stage of cancer gathered in the CARE PH database vis-à-vis databases from high-income-countries (HIC) like the United States, provides breast cancer screening programs in LMIC a good baseline upon which to assess the success of their cancer screening and early detection programs, with the goal being to diagnose more non-palpable breast masses in the earlier stages of cancer.

### Limitations of the HBCR app

The database, despite being equipped with fields for recording the stage at the time of diagnosis, the administered treatments, and the subsequent status of the diagnosed patients, largely lacks information in these categories, as depicted in Table 2. Among the modules comprising the hospital cancer registry application, it is observed that the module containing patient demographics exhibits the lowest incidence of missing data, ranging from 0% to 20%. In contrast, the module dedicated to capturing baseline characteristics of primary cancer displays a relatively higher degree of missing data spanning from 21% to 50%. The module concerning patient status or outcome after initial baseline data input records the highest incidence of missing data, with values ranging from 91% to 100%. Table 2 presents that only 25% of the cancer stage fields within the central database are completed, and a minimal 2% of the cancer status or outcome data fields are filled up.

The notable prevalence of missing data in the cancer registry stems from the intricacies of its data input process. Information pertaining to cancer staging and treatment outcomes is not readily available within documents found in catchment areas such as pathology reports, chemotherapy logbooks and radiation oncology reports, but is contained in either paper charts or electronic medical records (EMR). Remarkably, only 4 of the 44 (9%) participating CARE PH hospitals have EMR systems, and, regrettably, even in such instances, there is the absence of an application program interface (API) for seamless integration between the hospital’s EMR and the HBCR system. Although staging and treatment status can be determined by manual methods such as poring over paper records, cross-referencing with CT scan findings and laboratory reports via patient PIN numbers, or relying on physicians to enter staging data or patient status into the registry, these approaches prove inefficient, requiring additional manpower and funding. Moreover, it is to be noted that Doctor Champions and Tumor Registrars, who voluntarily engage in the HBCR system, may concurrently bear other responsibilities beyond data collection and encoding.

### Proposed framework of the HBCR app

The Philippine Health Insurance Corporation or Philhealth is mandated by the Universal Healthcare Act of 2019 to establish the National Health Data Repository (PHIC-NHDR). While the systems architectural design of the PHIC-NHDR starts with each hospital in the country transmitting their EMRs centrally via application programming interface or APIs, less than 60% of all hospitals in the country have EMRs.

To address this issue, CAREPH has devised an interim solution: to find a way to include staging and treatment data, which is the purview of physicians and not data encoders, a mandatory requirement in the monthly radiation oncology reports and chemotherapy logbooks. Such a step is particularly relevant for hospitals lacking EMRs at present. For those CARE PH hospital members already equipped with EMRs, the subsequent challenge lies in developing APIs that facilitate the seamless transfer of data from EMR systems to the CARE PH application, or alternatively, from the hospital information system (HIS) to the CARE PH application. Once done, the existing gaps in staging, treatment, and patient outcomes data will effectively be bridged, rendering the CARE PH HCBR system’s database will be more robust, informative, and useful.

More granular data collection particularly for common cancers like breast, lung, and colorectal cancer, i.e., identification of molecular or genomic biomarkers to be used as guide to treatment, can be included in future plans for HBCR, or for site-specific cancer registries. Once anonymized, such data no longer fall under the classification of human data and can be opened up for scientific research purposes. Researchers can then interrogate the data with relevant research questions, consequently leveraging big data analysis, machine learning, and artificial intelligence in moving forward towards better and more accessible cancer healthcare in the country.

## Conclusion and recommendations

The creation of a nationwide system of hospital-based cancer registries has important implications for cancer management, especially in resource limited low and middle income countries. This is a fact recognized by healthcare providers, given the steady growth in the number of hospitals contributing to the CARE PH HBCR system from 2017 to 2022. The higher the volume of data points, the clearer and more accurate the picture of the cancer burden in the country. Such data can now be used as a baseline for epidemiologic research studies, or for the assessment of the success of future intervention programs that aim to decrease the incidence of cancer by the promotion of cancer screening and prevention in the communities; or create better outcomes for patients by streamlining treatment protocols.

The challenges identified in this analysis include lack of baseline staging and first-line treatment data, and lack of status changes and subsequent treatment of the disease over time. Digital transformation of hospitals from paper-based charts to EMRs and the integration of the HBCR to the EMR and hospital information system (HIS) will likely be the best solution for these limitations.

The main lesson learned by CARE PH in the past 6 years is that all the elements of a proper HBCR–data privacy, quality, integrity, interoperability, adaptability, flexibility to modern technology–embedded in a well designed enterprise architecture, functioning under the guidance of a dedicated and strong leadership and governance team, must be present in order to create and maintain a robust HBCR that is useful for furthering registry and research in the country.

The researchers recommend that the creation and maintenance of HBCRs nationwide must be harmonized, not siloed, and must be embedded in all relevant national programs and legislations.
